# Inflammatory bowel disease and risk of Parkinson’s disease: evidence from a meta-analysis of 14 studies involving more than 13.4 million individuals

**DOI:** 10.3389/fmed.2023.1137366

**Published:** 2023-04-20

**Authors:** Hong-xing Li, Cui Zhang, Kai Zhang, Yi-zhe Liu, Xiao-xiao Peng, Qiang Zong

**Affiliations:** ^1^Department of Neurosurgery, Shengli Oilfield Central Hospital, Dongying, Shandong, China; ^2^Department of Otolaryngology, Shengli Oilfield Central Hospital, Dongying, Shandong, China; ^3^Department of Stomatology, Dongying District People’s Hospital, Dongying, Shandong, China

**Keywords:** Parkinson’s disease, inflammatory bowel disease, Crohn’s disease, ulcerative colitis, meta-analysis, risk factor

## Abstract

**Background:**

The relationship between inflammatory bowel disease (IBD) and the risk of Parkinson’s Disease (PD) has been investigated in several epidemiological studies. However, the results of these studies were inconclusive and inconsistent. We evaluated the potential relationship between IBD and PD risk by a meta-analysis.

**Methods:**

Search the electronic databases PubMed, Embase and Cochrane databases from inception to November 30, 2022, to identify relevant studies that assess the risk of PD in patients with IBD. The cohort, cross-sectional, mendelian randomization and case-control studies that reported risk estimates of PD and IBD were included in our analysis. The random-effect model and fixed-effects model were used to calculate the summary relative risks (RRs) with 95% confidence intervals (CIs).

**Results:**

In total, 14 studies (nine cohort studies, two cross-sectional studies, two mendelian randomization studies and one case-control study) involving more than 13.4 million individuals were analyzed in our analysis. Our results suggested that the risk of PD in IBD patients is moderately increased, with the pooled RR was 1.17 (95% CI: 1.03–1.33, *P* = 0.019). Omit of any single study from this analysis had little effect on the combined risk estimate. No evidence of publication bias was found. In the subgroup analysis, the combined RR was 1.04 (95% CI: 0.96, 1.12, *P* = 0.311) for Crohn’s disease (CD), and 1.18 (95% CI: 1.06, 1.31, *P* = 0.002) for ulcerative colitis (UC). In addition, a significant association was identified in patients with IBD aged ≥ 60 years (RR = 1.22; 95% CI: 1.06–1.41, *P* = 0.007), but not in age < 60 years (RR = 1.19; 95% CI: 0.58–2.41, *P* = 0.639). Meanwhile, the meta-analysis results suggested a protective role for IBD medication use against PD development, with the RR was 0.88 (95% CI: 0.74, 1.04, *P* = 0.126).

**Conclusion:**

Our results indicated that patients with IBD had a moderately higher risk of PD compared to non-IBD individuals. Patients with IBD should be aware of the potential risks for PD, especially who were ≥ 60 years old.

## 1. Introduction

Parkinson’s disease (PD), a multifactorial movement disorder disease, which is the second most common neurodegenerative disorder. The primary cause of this disease is the loss of dopaminergic neurons in the substantia nigra and the abnormal accumulation of intracellular α-synuclein, which usually develops between 65 and 70 years old, and accounts for up to 1% of people over 60 years old (1). The disease progresses slowly and the typically clinical motor symptoms, including rigidity, bradykinesia, stooping posture, and resting tremors, as well as non-motor features such as hyposmia, depression, anxiety, sleep disturbances, and cognitive decline (2). The cause of PD is unclear, except for a small number of young cases caused by rare genetic mutations, the risk of developing PD is the complex interaction of genetic and environmental factors (3). Recently, more and more evidence show that systemic inflammation is closely related to the pathogenesis of PD, which suggests that chronic inflammation of peripheral organs may lead to neurodegeneration of PD by changing the permeability of the blood-brain barrier (4, 5), and the intestinal inflammation may play an essential role in the pathogenesis of PD (6, 7).

Inflammatory bowel disease (IBD) is chronic systemic inflammatory disorders, which including Ulcerative colitis (UC) and Crohn’s disease (CD). It has been reported that more than 1.8 million adults are affected by IBD in the United States (8), recurrent abdominal pain, rectal bleeding, diarrhea, weight loss, or anemia are the main clinical manifestation. Generally, CD involves the ileum, colon, or any region of the intestine, whereas UC only involves the rectum to the pancolitis and is limited to the mucosa. IBD mainly affects the gastrointestinal tract, but it may also be related to systemic inflammation and extraintestinal manifestations (9).

More and more people realize that the intestinal environment and the central nervous system communicate through the activities of the gut-brain axis, which leads to the hypothesis that chronic intestinal inflammation may lead to neurodegeneration in PD (6). Epidemiological evidence also suggests that IBD may be associated with PD risk, but so far, the results are not consistent (10–22). A meta-analysis of four studies was conducted by Zhu et al. (23) showed an increased risk of PD in the IBD population with the relative risk (RR) was 1.41 (95% CI:1.19, 1.66). Szandruk-Bender et al. (24) conducted a meta-analysis that showed a higher risk of PD among IBD patients compared to the non-IBD patients, but only four studies (cohort study) were designed to assess the relationship between PD and IBD in their analysis. Another a meta-analysis was conducted by Zhu et al. (25) demonstrated that the overall risk of PD was significantly higher in IBD patients than in the general population, with the RR was 1.24 (95% CI:1.15, 1.34, and *P* < 0.001). In recent years, several clinical studies with large samples have been conducted to investigate the association between IBD and PD, again, with conflicting results (10, 11, 18, 19, 26, 27). In these studies, two (cohort study) reported positive relationships (10, 19), while three (two mendelian randomization studies and one cohort study) showed no association (18, 26, 27) and one (cross-sectional study) found an inverse relationship (10). Currently, the effects of IBD on PD risk are still controversial. Therefore, it is appropriate that an updated meta-analysis was conducted to clarify this issue better. In this meta-analysis, the primary aim was to investigate whether IBD patients have a higher risk of PD. Meanwhile, through this study, we will research whether early IBD medication use could reduce the risk of PD. PD is a common disease that affects about 6.1 million people worldwide and has a great impact on society (28). Understanding the relationship between IBD and PD may find new approaches to the prevention and diagnosis of PD and improve the effectiveness of its treatment.

## 2. Materials and methods

### 2.1. Literature search

The PubMed, Embase and Cochrane databases were systematically searched to identify relevant publications from the inception of the databases to November 30, 2022, by two authors (HXL and CZ). The research strategy was as following: “(Inflammatory bowel disease OR Crohn’s disease OR Ulcerative colitis OR IBD OR Bowel Diseases, Inflammatory) and (Paralysis agitans OR Parkinson disease OR Parkinson OR Parkinson’s OR Parkinson’s disease OR PD).” Additionally, we also checked the reference list of related articles and reviewed them to identify other potential studies.

### 2.2. Inclusion and exclusion criteria

A study was considered eligible in this meta-analysis if it met the following criteria: (1) case-control, cohort, or cross-sectional study that explored the association of IBD and PD risk; (2) the publication was written in English; (3) IBD, UC or CD was the exposure, and PD was the outcome of interest; (4) the RR, odds ratio (OR), hazard ratio (HR), standardized incidence ratios (SIR), or incidence rate ratio (IRR), and their 95% confidence interval (CI) were reported, or could be calculated from the original data. If the same study (or the same data source) has more than one publication, only the study with the largest number of samples is included. Studies were excluded if they were cases report, commentaries, reviews, comments, editorials, or experimental studies.

### 2.3. Data extraction and quality assessment

For each study, the following data were extracted: first author’s name, year of publication, study location/period, data source, study design, sample size, follow-up duration, age, variables adjustment, risk estimates, and their corresponding 95% CIs. The Newcastle-Ottawa Scale (NOS) was used to assess the quality of cohort study (29), and the total score ≥ 7 stars was defined as a high-quality study. The quality of the cross-sectional study was assessed by an 11-item checklist, which was recommended by Agency for Healthcare Research and Quality (AHRQ), and defined as low quality (0–3), moderate quality (4–7), or high quality (8–11) (30).

All data were collected and processed by two independent researchers (CZ and XXP), and any disagreements were resolved through discussion and consensus with the third investigator (QZ).

### 2.4. Statistical analysis

The standard criteria of observational researches were performed and reported in our meta-analysis (31). All statistical analyses were conducted using STATA 12.0 statistical software (Stata Corporation, College Station, TX, USA), and all statistical tests were two-sided with P ≤ 0.05 indicating statistical significance. Since the absolute risk of PD is low, HR, OR, and IRR are approximate equivalent to RR (32). Therefore, in the current study, RR was used as an indicator to measure the relationship between IBD and PD risk. The heterogeneity between the included studies by Cochran’s Q statistic (33) and I^2^ statistic (34), and defined as low heterogeneity (*I*^2^ ≤ 25%), moderate heterogeneity (*I*^2^ = 25–50%), or high heterogeneity (*I*^2^ > 50%). RR was calculated using a random-effects model (DerSimonian and Laird), which was considered as a conservative method of estimating cumulative effects. To explore the impact of a single study on the overall risk estimates of IBD and PD risk, we conducted a sensitivity analysis by omitting each study in each turn (35). Begg’s funnel plots (36) and Egger’s regression test (37) were conducted to assess publication bias. Since rather small numbers of studies for variables “CD and UC,” only variable “IBD” was performed for sensitivity analysis and publication bias.

To explore potential heterogeneity and further clarify the correlation between IBD and PD risk, several additional subgroups were carried out according to study design (cohort vs. cross-sectional vs. mendelian randomization vs. case-control study), disease subtype (CD vs. UC), gender (male vs. female), age (<60 vs. <65 vs. ≥60 years) and IBD treatment (e.g., anti-tumor necrosis factor, 5-aminosalicylic acid, immunomodulators, and IBD-related bowel surgery).

## 3. Results

### 3.1. Search results and characteristics of studies

There were 785 potentially relevant publications from PubMed (*n* = 319), Embase (*n* = 445) and Cochrane databases (*n* = 21) were initial screening identified. After reading the title or abstract, 765 articles were excluded, and the full-texts of the 20 potentially relevant publications were then scrutinized. When manually searching the references of all relevant studies, one article was included (17).

**FIGURE 1:**
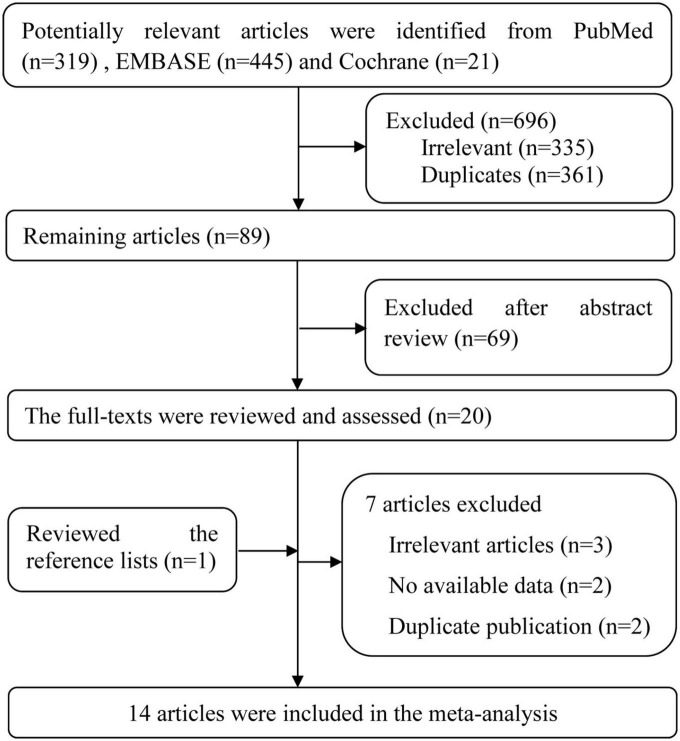
Flow chart of the literature search and study selection for relevant studies in the meta-analysis.

After full-texts reviewed, seven articles were excluded. The excluded reasons are as follows: three studies were irrelevant reports (7, 38, 39), two articles did not provide available data (40, 41), four publications were from two same researches and same data source (12, 14, 16, 17), so only two most complete information articles were included (12, 14). Finally, 14 eligible studies (nine cohort studies, two cross-sectional studies, two mendelian randomization studies and one case-control study) met the inclusion criteria and were incorporated into our analysis. The flow chart of the literature search and study selection for relevant studies is detailed in Figure 1.

The main features of the included studies were summarized in Table 1. Of the 14 studies included, nine were cohort studies (11–15, 18–21), two cross-sectional studies (10, 22), two mendelian randomization studies (26, 27) and the remaining one was a case-control study (16). Altogether, these articles were published between 2011 and 2022, involving more than 13.4 million individuals. The study location was from Chinese mainland (27), Taiwan (15, 21), Germany (26), United States (14, 16, 18, 20), Swedish (13, 22), Danish (12), Korean (11, 19), and Spain (10). Among the 14 studies, HR was used in six studies (11–13, 15, 18, 19), OR was used in six studies (10, 16, 21, 22, 26, 27), while the remaining two studies used IRR (14) and SIR (20), respectively. Two studies of the type Mendelian randomization cannot be evaluated (26, 27), and the remaining studies were scored ≥ 8 on the NOS and AHRQ, meaning that the studies were of high quality.

**TABLE 1:** Study characteristics of inflammatory bowel disease and the risk of Parkinson’s disease.

References	Study location/ Period	Data source	Study design	Sample size	Follow-up (Y)	Age (Y)	IBD variables assessed and adjustment	Risk estimate: adjusted effect variable (95% CI)	Quality assessment scale of the studies
Freuer and Meisinger (26)	German N/P	GWAS of the UK Biobank	Mendelian randomization	7,045 (IBD); 456,327 (Non-IBD); 56,306 (PD); 1,400,000 (Non-PD)	N/A	N/P	IBD, CD, UC; N/P (Adjustment)	OR IBD: 0.98 (0.93, 1.04) CD: 1.00 (0.98, 1.03) UC: 1.02 (0.98, 1.06)	N/A
Li and Wen (27)	China N/P	European GWAS	Mendelian randomization	25,683 (IBD); 31,954 (Non-IBD); 33,674 (PD); 449,056 (Non-PD)	N/A	N/P	CD, UC; N/P (Adjustment)	OR CD: 1.01 (0.97, 1.05) UC: 1.01 (0.96, 1.06)	N/A
Coates et al. (18)	USA 2005.01–2010.12	United States MCCED	Retrospective cohort	154,051 (IBD); 154,051 (Non-IBD)	3.8	18–64	IBD, CD, UC; Adjusted for age, sex, residence type, US region, comorbidities, and behavior	HR IBD: 1.01 (0.72, 1.42) CD: 1.33 (0.80, 2.21) UC: 0.81 (0.51, 1.29)	NOS = 9
Kim et al. (19)	Korean 2009–2011	Korea NHIS	Retrospective cohort	24,830 (IBD); 99,320 (Non-IBD)	5 (Longest)	≥40	IBD, CD, UC, sex, age; Adjusted for age, sex, residential area, and comorbidities	HR IBD: 1.56 (1.24, 1.97) CD: 1.03 (0.58, 1.84) UC: 1.69 (1.32, 2.15)	NOS = 9
Pinel Ríos et al. (10)	Spain 2014.12	APHS	Cross-sectional	19,966 (PD); 7,485 (IBD);	N/A	≥50	IBD, age; Adjusted for age and sex	OR IBD: 0.94 (0.72, 1.23)	AHRQ = 10
Park et al. (11)	Korean 2010.01–2013.12	Korea NHIS	Retrospective cohort	38,861 (IBD); 116,583 (Non-IBD)	4.9 (Mean)	39.9 (Mean)	IBD, CD, UC, age, sex; Adjusted for age, sex, place of residence, income level, comorbidities	HR IBD: 1.95 (1.49, 2.54) CD: 2.44 (1.22, 4.86) UC: 1.92 (1.43, 2.56)	NOS = 8
Villumsen et al. (12)	Danish 1977–2014	Danish NPR	Retrospective cohort	76,477 (IBD); 7,548,259 (Non-IBD)	22 (Longest)	≥15	IBD, CD, UC, sex, age; Adjusted for Charlson Comorbidity Index	HR IBD: 1.22 (1.09, 1.35) CD: 1.12 (0.89, 1.40) UC: 1.35 (1.20, 1.52)	NOS = 9
Bähler et al. (22)	Switzerland 2014	Switzerland HIG	Cross-sectional	4,791 (IBD); 1,114,638 (Non-IBD)	N/A	> 0	IBD; Adjusted for age, sex, language area, type of insurance coverage, and urbanization	OR IBD: 0.92 (0.67, 1.27)	AHRQ = 10
Weimers et al. (13)	Swedish 2002.01–2014.12	Sweden NPR	Retrospective cohort	39,652 (IBD); 396,520 (Non-IBD)	249,784,4 (Person-years)	≥50	IBD, CD, UC, age, sex; Adjusted for sex, age, index date, and place of residency	HR IBD: 1.30 (1.00, 1.60) CD: 1.10 (0.70, 1.70) UC: 1.30 (1.00, 1.70)	NOS = 9
Peter et al. (14)	United States 2000.01–2016.03	United States THMCD + MSD	Retrospective cohort	144,018 (IBD); 720,090 (Non-IBD)	≥0.5	≥18	IBD, CD, UC; Adjusted for time-varying age group and sex, and offset by time.	IRR IBD: 1.28 (1.14, 1.44) CD: 1.26 (1.03, 1.53) UC: 1.31 (1.14, 1.51)	NOS = 9
Camacho-Soto et al. (16)	United States 2004–2009	USA Medicare base file (BASF)	Case-control	89,790 (PD); 118,095 (Non-PD)	N/P	>65	IBD, CD, UC, age; Adjusted for age, race, sex, and probability of smoking, comorbidities	OR IBD: 0.85 (0.80, 0.91) CD: 0.83 (0.74, 0.93) UC: 0.88 (0.82, 0.96)	NOS = 7
Lin et al. (15)	Taiwan 2000–2011	China Taiwan LHID 2000	Retrospective cohort	8,377 (IBD); 33,492 (Non-IBD)	7.30 (IBD); 7.13 (No-IBD)	≥20	IBD, CD, UC, age, sex; Adjusted for age, sex, and comorbidities	HR IBD: 1.35 (1.08, 1.68) CD: 1.40 (1.11, 1.77) UC: 0.94 (0.49, 1.84)	NOS = 9
Hsu et al. (21)	Taiwan 2000–2008	China Taiwan NHRI	Retrospective cohort	1,698 (PD); 6,792 (Non-PD)	57,617 (Person-years)	>0	IBD; N/P (Adjustment)	OR IBD: 1.11 (0.78, 1.60)	NOS = 7
Li et al. (20)	USA 1964.01–2007.12	Sweden PHCRCMD	Retrospective cohort	30,631 (IBD); N/P (Non-IBD)	43 (Longest)	>0	CD, UC, age; Adjusted for age, period, socioeconomic status, region of residence, hospitalization of COPD, and alcoholism and alcohol-related liver disease	SIR CD: 0.62 (0.33, 1.07) UC: 1.23 (0.90, 1.64)	NOS = 8

IBD, inflammatory bowel disease; PD, Parkinson’s disease; CD, Crohn’s disease; UC, ulcerative colitis; OR, odds ratio; HR, hazard ratio; IRR, the incidence rate ratio; Y, year; CI, confidence interval; NOS, Newcastle-Ottawa Scale; AHRQ, Agency for Healthcare Research and Quality; N/A, not applicable; N/P, not provided; GWAS, Genome-wide association study; UK, The United Kingdom; MCCED, the marketScan commercial claims and encounters database (Truven Health Analytics); APHS, Andalusian public health system; NHIS, national health insurance service; HIG, the helsana insurance group; NPR, national patient register; THMCD, the truven health marketScan commercial database; MSD, the medicare supplemental database; LHID 2000, longitudinal health insurance database 2000; NHRI, the national health research institutes; PHCRCMD, the primary health care research center migMed database; SIR, standardized incidence ratios.

### 3.2. Inflammatory bowel disease and Parkinson’s disease risk

Twelve studies (10–16, 18, 19, 21, 22, 26) estimated the association between IBD and PD risk, with the overall pooled RR was 1.17 (95% CI: 1.03–1.33, and *P* = 0.019). Although significant heterogeneity was found among these 12 studies (*P* = 0.000, *I*^2^ = 89.5%), the result suggested that patients with IBD had a 17% higher risk of PD risk compared with non-IBD.

**FIGURE 2:**
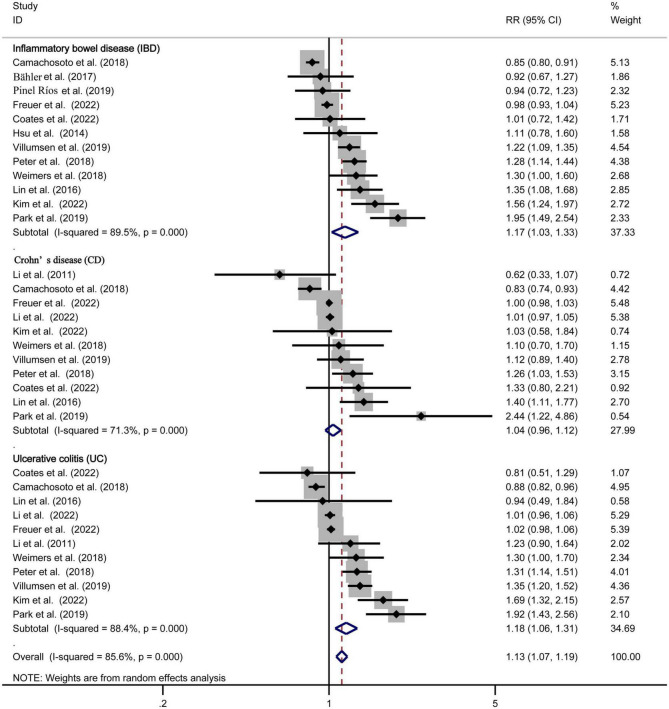
Forest plot of the Parkinson’s disease risk in patients with inflammatory bowel disease, Crohn’s disease, and ulcerative colitis.

The risk estimates for CD and UC were reported in 11 studies (11–16, 18–20, 26, 27), with the combined RR for CD was 1.04 (95% CI: 0.96, 1.12, and *P* = 0.311), for UC was 1.18 (95% CI: 1.06, 1.31, and *P* = 0.002). Meanwhile, the significant heterogeneity was detected in the analysis, with (*P* = 0.000, *I*^2^ = 71.3%) and (*P* = 0.000, *I*^2^ = 88.4%) respectively. The forest plot of the PD risk in patients with IBD, CD, and UC are shown in Figure 2.

### 3.3. Stratified analysis

Further subgroup analyses were performed to expose the potential heterogeneity, and the results are shown in Table 2.

**TABLE 2:** Results of meta-analysis for inflammatory bowel disease and the risk of Parkinson’s disease.

Group	No. of studies	RR (95% CI)	*P*-value	*I*^2^ (%)	*P*-heterogeneity	Analysis model
IBD	12	1.17 (1.03, 1.33)	0.019	89.5	0.000	Random-effects model
**Study design**						
Cohort study	9	1.32 (1.18, 1.44)	0.000	52.0	0.034	Random-effects model
Cross-sectional study	2	0.93 (0.76, 1.14)	0.499	0.0	0.919	Fixed-effects model
Mendelian randomization study	2	1.00 (0.98, 1.03)	0.805	0.0	0.351	Fixed-effects model
Case-control study	1	–	–	–	–	–
**Disease subtype**						
CD	11	1.04 (0.96, 1.12)	0.311	71.3	0.000	Random-effects model
UC	11	1.18 (1.06, 1.31)	0.002	88.4	0.000	Random-effects model
**Gender**						
Male	5	1.27 (1.15, 1.41)	0.000	0.0	0.577	Fixed-effects model
Female	5	1.54 (1.19, 1.98)	0.001	74.4	0.004	Random-effects model
**Age (years)**						
<60	3	1.19 (0.58, 2.41)	0.639	75.3	0.017	Random-effects model
<65	7	1.09 (0.72, 1.66)	0.681	81.4	0.000	Random-effects model
≥60	8	1.22 (1.06, 1.41)	0.007	51.8	0.053	Random-effects model
IBD medication use	6	0.88 (0.74, 1.04)	0.126	59.8	0.029	Random-effects model
IBD-related bowel surgery	2	0.58 (0.28, 0.89)	0.013	77.4	0.109	Random-effects model

IBD, inflammatory bowel disease; PD, Parkinson’s disease; CD, Crohn’s disease; UC, ulcerative colitis.

In the stratified analysis by study design, 9 cohort studies (11–15, 18–21) were included to examine the PD risk. The result found that the IBD patients had an increased risk of PD by nearly 32% compared with non-IBD patients, with a pooled RR was 1.32 (95% CI: 1.18, 1.44, *P* = 0.000). Two cross-sectional studies (10, 22) and two mendelian randomization studies (26, 27) were included to examine the PD risk, with the pooled RR were 0.93 (95% CI: 0.76, 1.14, *P* = 0.499) and 1.00 (95% CI: 0.98, 1.03, and *P* = 0.805), respectively. These results showed that IBD patients do not increase the risk of PD compared with non-IBD patients. For the remaining one case-control study (16), we did not conduct a meta-analysis.

In the stratified analysis by gender, five studies were included (11–13, 15, 19). The subgroup analyses revealed an increased risk of PD in both male and female IBD patients. The pooled RR for male was 1.27 (95% CI: 1.15, 1.41, and *P* = 0.000) with low heterogeneity (*I^2^* = 0.0%, *P* = 0.577), and the RR for female was 1.54 (95% CI: 1.19, 1.98, and *P* = 0.001) with high heterogeneity (*I^2^* = 74.4%, *P* = 0.004).

In the stratified analysis by age, a total of eight studies providing sufficient data in our analysis (10–13, 15, 16, 19, 20). Among the eight studies, three (11–13) evaluated the association between IBD and PD risk at age < 60, with the summary RR was 1.19 (95% CI: 0.58, 2.41, and *P* = 0.639). However, eight studies evaluated the association in age ≥ 60, with the summary RR was 1.22 (95% CI: 1.06, 1.41, and *P* = 0.007). We observed a 22% increased risk of PD in patients aged ≥ 60 years.

In the stratified analysis by IBD medication use, six studies (10, 11, 13, 14, 16, 19) providing sufficient data to assess the relationship between the risk of PD and patients with IBD undergoing treatment. The results suggested a protective role for IBD medication use against PD development, with the RR was 0.88 (95% CI: 0.74, 1.04, and *P* = 0.126).

In the stratified analysis by IBD-related bowel surgery, two studies (13, 16) providing sufficient data to assess the relationship between the risk of PD and patients with IBD undergoing surgery. The results suggested a protective role for IBD-related bowel surgery against PD development, with the RR was 0.58 (95% CI: 0.28, 0.89, and *P* = 0.013).

### 3.4. Sensitivity analysis and publication bias

**FIGURE 3:**
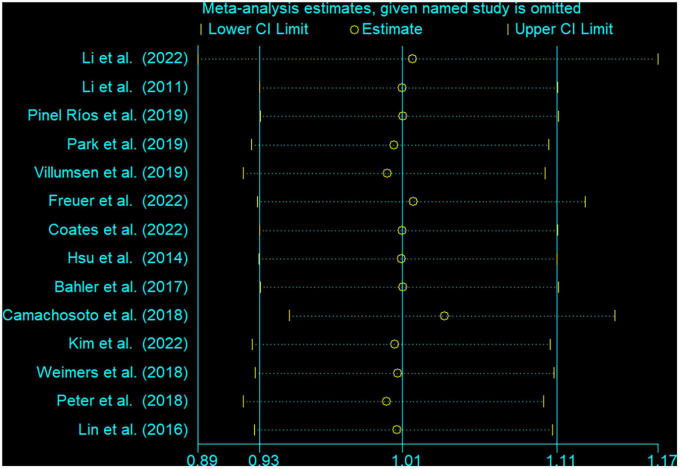
Sensitivity analysis for the association between inflammatory bowel disease and the risk of Parkinson’s disease. The two ends of the lines represent the 95% CI.

**FIGURE 4:**
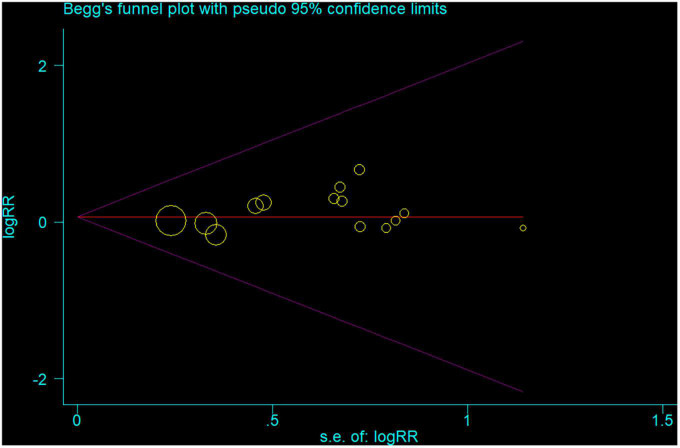
Begg’s funnel plot of all 14 studies the associations between inflammatory bowel disease and the risk of Parkinson’s disease. Each point represents separate study for the indicated association.

In the sensitivity analysis, the pooled RR with a narrow range from 1.01 (95% CI: 0.92, 1.10) to 1.04 (95% CI: 0.95, 1.14) (Figure 3). The Begg’s funnel plot and Egger’s regression test were used to assess potential publication bias in our study. No evidence of publication bias was detected, for Begg’s (*P* = 0.622) and Egger’s (*P* = 0.404) (Figure 4).

## 4. Discussion

To our knowledge, this is the most comprehensive meta-analysis and systematic review investigating the association between IBD and PD risk up to date. In this meta-analysis, although only 14 studies, the study involving more than 13.4 million subjects. Our study demonstrated that the risk of PD in IBD patients was an increase of 17% in a comparison of patients with non-IBD. Meanwhile, subgroup analysis also found that IBD patients had an increased risk of developing PD regardless of stratification factors, except for CD group and age < 60 years. Furthermore, the study also hinted early IBD medication use in patients with IBD may reduce the risk of PD.

Subgroup analysis by age in our study demonstrated a small but statistically significant trend toward an increased risk of PD in IBD patient age ≥ 60 years (RR = 1.22; 95% CI: 1.06–1.41; *P* = 0.007), but not in age < 60 years old (RR = 1.19; 95% CI: 0.58–2.41; *P* = 0.639). Weimers et al. (13) found that 80% of PD events occurred in patients with IBD ≥ 60 years. However, Villumsen et al. (12) reported a cohort of 8.8 million individuals in 2018, with follow-up more than 20 years, who found an increased risk of PD independent of age at IBD diagnosis. The PD risk was similar in patients with IBD diagnosed at age < 40 years, 40—65 years, and >65 years, with the HR were 1.30 (95% CI: 1.19–1.52), 1.25 (95% CI: 1.06–1.47), and 1.22 (95% CI, 0.82–1.82), respectively, (12). Age is a crucial risk factor for the development of PD (42). According to the epidemiological survey, PD affects 1–2 per 1,000 of the population, which is rare before the age of 50 years, the incidence rate of PD in population over 60 is about 1%, and most PD patients are diagnosed from 65 to 70 years old (1). In any study, the older the baseline age, the higher the risk of PD. Our study included a large number of individuals, and the larger the number, the closer to the results of epidemiology. Although we utilized the random-effects model to estimate cumulative effects, those results are not robust. Therefore, it is necessary to further explore this issue.

In a meta-analysis, heterogeneity should be concerned. Moderate and high heterogeneity was observed in our study. Low heterogeneity was observed from a subgroup analysis of male, with heterogeneity (*I^2^* = 0.0%, *P* = 0.577). It should not be surprising that a meta-analysis exhibits high heterogeneity. These 14 studies were included in our analysis were all derived from different research designs, geographic regions, demographic characteristics, and adjustments for confounding factors, which may be the sources of heterogeneity. Although we conducted a subgroup analysis of the study design, people from different geographical regions have different living habits, work styles, health care visit, and genetic backgrounds, which may lead to a high level of heterogeneity. Therefore, we used the random-effects model in these analyses with high heterogeneity, which was considered as a conservative method of estimating cumulative effects.

The exact mechanism of the association between IBD and PD risk is still unknown. On the one hand, more and more evidences support that gastrointestinal tract inflammatory plays a crucial role in the initiation and progression of PD (7, 41, 43). In PD patients, gastrointestinal symptoms may precede PD motor symptoms for many years, and these patients may exhibit some inflammation response associated with a-synuclein accumulation in the gastrointestinal tract (44). The Gut-brain axis is a link between the enteric and the central nervous system, which is used for bidirectional communication between the two (7, 45). Some gastrointestinal tract microbial components may cause intestinal inflammation, which could regulate and promote the pathways of α-synuclein aggregation (46). The deposition of α-synuclein in the bowel wall may diffuse through the vagus nerve, and leading dopaminergic degeneration in PD patients (46). In the pathogenesis of IBD and PD, there are some similar pro-inflammatory factors in the inflammatory process of both diseases, such as TNF-α and IL-1β. Dopaminergic degeneration of central substantia nigra striatum with intestinal inflammation is related to the increase of TNF-α and IL-1β (47). Peter et al. (14) reported an interesting American cohort study that the incidence rate of PD in patients with IBD treated with anti-TNF was reduced by 78% compared with those without exposure, which supported the role of systemic inflammation in the pathogenesis of both IBD and PD. Camacho-Soto et al. (16) reported a case-control study from the United States, the group reported an inverse association between immunosuppressant and steroid use and development of PD. A Swedish cohort study of IBD patients investigating the risk of PD with regards to IBD medication use, IBD patients never exposed to thiopurines or anti-tumor necrosis factor were 60% more likely to increase risk of developing PD, with the HR was 1.60 (95% CI: 1.2–2.2) (13). A Korean population-based study was conducted by Park et al. (11), this study showed that corticosteroid use as a preventive effect on the risk of PD in patients with CD. Meanwhile, during 9,950 person-years, among 2,110 patients who received anti-tumor necrosis factor agents, no patient experienced PD. These findings indicate that IBD medication use is associated with a reduced risk of PD, which is basically consistent with our meta-analysis. Therefore, aggressive treatment may reduce the risk of PD.

On the other hand, growing research shows that PD and IBD have common key genetic factors. Bialecka et al. (48) published the first article on a possible genetic relationship between IBD and PD in 2007. According to previous studies, IBD and PD share a common genetic risk profile, such as NOD2, LRRK2, and MAPT genes (7, 49–51). For example, LRRK2 was initially identified as the causative gene of PD, but recently it has been associated with the increased incidence rate of CD (52–54). However, two recent studies utilized the Mendelian randomization analysis to investigate the causal relationship between genetically predicted IBD and the risk of PD, these studies provided no evidence that genetically predicted IBD are causally related to PD (26, 27). Therefore, both active peripheral inflammation and genetic risk may be closely related to PD and IBD, and there may be a link between the two diseases. But this association should be clarified by more epidemiological studies.

Several strengths should be mentioned in our study. Firstly, our study included 14 studies involving a large number of participants, which improved the ability to find significant associations and provided more reliable estimates. Compare with the former meta-analysis in 2022 by Zhu et al. (25) and Szandruk-Bender et al. (24), more individuals participated and more subgroups were analyzed. Secondly, some subgroup analyses were carried out to assess the accuracy and reliability of our results. Thirdly, the originally included studies were cohort, cross-sectional, mendelian randomization and case-control study design, in which the recall and selection biases could be significantly reduced. In addition, sensitivity analysis showed that excluding any single study had little effect on the overall risk estimation. All these measures support the robustness of the findings.

Some potential limitations in the current study should be considered. First, although a large number of participants were involving, only 14 studies were included in the study. Second, only English publications were included, so that other language studies may be ignored. Third, moderate to high heterogeneity was observed in our study, although several subgroups were conducted, it may be inevitable, because the confounding factors from original studies were unavoidable.

## 5. Conclusion

In conclusion, this meta-analysis provides evidence that IBD was associated with an increased risk of PD. IBD patients should be paid more attention to the potential risk of PD, especially those over 60 years old. In addition, early IBD medication use in patients with IBD might reduce the risk of PD development. Further prospective larger well-designed epidemiological studies are warranted to validate this finding from our meta-analysis.

## Data availability statement

The raw data supporting the conclusions of this article will be made available by the authors, without undue reservation.

## Author contributions

H-XL and QZ conceived and designed the work. H-XL, X-XP, and CZ contributed to the systematic literature search, selected the studies for inclusion, and extracted the data. KZ, X-XP, and CZ analyzed and interpreted the data and made the figures. Y-ZL, KZ, and QZ wrote, reviewed, and critiqued the manuscript. All the authors revised the important sections in the manuscript and approved the final draft.
